# Pre-induction LDH as a prognostic factor for outcome of high dose chemotherapy (HDCT) for germ cell tumours relapsing or refractory to conventional chemotherapy

**DOI:** 10.1054/bjoc.1999.1140

**Published:** 2000-05-18

**Authors:** J Shamash, R T D Oliver, C J Gallagher, A C Newland, T A Lister, S Kelsey, R K Gupta, C A O'Doherty

**Affiliations:** St Bartholomew's Hospital, Theatre D, 1st Floor King George V Building, West Smithfield, London, EC1A 7BE, UK


*British Journal of Cancer* (2000) **82**(12), 2022–2023
© 2000 Cancer Research Campaign
doi: 10.1054/ bjoc.1999.1140, available online at http://www.idealibrary.com on IDEAL®

Letters to the Editor

### Pre-induction LDH as a prognostic factor for outcome of high dose chemotherapy (HDCT) for germ cell tumours relapsing or refractory to conventional chemotherapy

**Sir**

To date, the prognostic index derived by Beyer et al (1996) which identifies disease site at presentation, level of hCG prior to HDCT and sensitivity to last cisplatin-based conventional chemotherapy regimen has proved to be the most useful way of predicting progression-free and overall survival following HDCT.

High-dose chemotherapy is most frequently used as consolidation following induction therapy; however, few publications have examined the relevance of patient therapy at predicting response prior to induction. At this centre the policy has been to give 3 cycles of cisplatin/ifosfamide-based induction therapy (for doses see Table 1) followed by high-dose chemotherapy.

The results of our last 30 consecutive patients have recently been reviewed and are presented in Table 1. All the patients were scored according to the system derived by Beyer et al (1996) and the results are in very close agreement with those predicted. Most patients were scored as zero (chemo-sensitive, non-mediastinal primary). However, in addition, several observations were made which have not been previously reported.

A raised lactate dehydrogenase (LDH) prior to induction chemotherapy predicted a poor outcome (only 1/10 progression free at 2 years versus 11/15 if normal, $P = 0.049$). The predictive value of this exceeds that of conventional tumour markers. Two therapeutic manoeuvres – namely the introduction of paclitaxel into induction therapy (TIP) and the dosing of carboplatin using AUC rather than mg $m^{-2}$ in combination with etoposide and an oxazophosphorine – were also examined. Paclitaxel did not appear to have improved outcome. Initially as part of the high-dose regimen patients received carboplatin with dosage calculated on the basis of mg $m^{-2}$. Subsequently dosing was changed to one using an AUC formula (Calvert et al, 1989). Recalculating the dosage to express it as an AUC was possible since patients had an EDTA clearance prior to HDCT. This enabled examination of the impact of the carboplatin AUC dose. There was no suggestion that higher doses of carboplatin were more effective.

We believe the observation of a raised LDH at induction carrying a poor prognosis needs to be validated, as the test is simple and in first line therapy increasingly recognized to be as good if not better than hCG as prognostic factor outcome (Mead

**Table 1** Pre-induction chemotherapy characteristics proceeding to high-dose chemotherapy

| | | No. of cases | Progression-free following HDCT | | |
|---|---|---|---|---|---|
| HCG (iu $ml^{-1}$) | < 1000 | 10 | 8 | $\chi^2 = 2.95$ | $P = 0.229$ |
| | ≥ 1000 | 9 | 4 | | |
| | Normal | 11 | 2 | | |
| AFP (ng $ml^{-1}$) | < 500 | 6 | 1 | $\chi^2 = 1.35$ | $P = 0.509$ |
| | ≥ 500 | 5 | 2 | | |
| | Normal | 19 | 11 | | |
| LDH (iu) | Normal | 15 | 11 | $\chi^2 = 3.89$ | $P = 0.049$ |
| | Raised | 10 | 1 | | |
| | Not available | 5 | 2 | | |
| No of conventional therapies pre-HDCT | 2 | 8 | 3 | $\chi^2 = 0.285$ | $P = 0.867$ |
| | 3 | 19 | 10 | | |
| | 4 or more | 3 | 11 | | |
| Prognostic scores | 0 | 24 | 12 | $\chi^2 = 2.51$ | $P = 0.284$ |
| | 1 | 2 | 1 | | |
| | 2 | 4 | 0 | | |
| Induction chemotherapy | | | | | |
| TIP[a] | | 13 | 6 | $\chi^2 = 1.41$ | $P = 0.494$ |
| VIP[b] | | 14 | 7 | | |
| Other | | 3 | 0 | | |
| Carboplatin dose by AUC | < 20 | 17 | 9 | $\chi^2 = 0.13$ | $P = 0.722$ |
| | ≥ 20 | 12 | 5 | | |

[a]TIP – paclitaxel, 210 mg $m^{-2}$, ifosfamide 4.5 g $m^{-2}$, cisplatin 100 mg $m^{-2}$. [b]VIP – etoposide 360 mg $m^{-2}$, ifosfamide 6 g $m^{-2}$, cisplatin 100 mg $m^{-2}$. HDCT – carboplatin 1200 mg $m^{-2}$ or AUC 20, etoposide 1500 mg $m^{-2}$ and either cyclophosphamide 6 g $m^{-2}$ or ifosfamide 9 g $m^{-2}$.

and Stenning, 1997). Identification of patients at an earlier stage, prior to induction therapy may allow patients predicted to do badly with cisplatin/ifosfamide based therapy to receive alternative experimental approaches.

*J Shamash, RTD Oliver, CJ Gallagher, AC Newland, TA Lister,*
*S Kelsey, RK Gupta and CA O'Doherty*
*St Bartholomew's Hospital, Theatre D, 1st Floor*
*King George V Building, West Smithfield, London EC1A 7BE, UK*

**REFERENCES**

Beyer J, Kramar A, Mandanas R, Linkesch W, Greinix A, Droz JP, et al (1996) High-dose chemotherapy as salvage treatment in germ cell tumors: a multivariate analysis of prognostic variables. *J Clin Oncol* **14**: 2638–2645

Calvert AH, Newell DR and Gumbrell LA (1984) Carboplatin dosage: prospective evaluation of a simple formula based on renal function. *J Clin Oncol* **7**: 1748–1756

Mead GM and Stenning S (1997) International germ cell cancer collaborative group prognostic factor scoring system. *J Clin Oncol* **15**: 594–603

doi: 10.1054/ bjoc.1999.1137, available online at http://www.idealibrary.com on IDEAL®

### A possible cause of testicular cancer

**Sir**

Fosså and Kravdal (2000) found that men with testicular cancer were subfertile both before and after diagnosis. Møller (1998) reported that men with testicular cancer sired significant excesses of daughters both before and after diagnosis. Fosså and Kravdal (2000) wrote that 'the low fertility after diagnosis may be partly due to the continuing inherent influence of a sub- or in-fecundity that also had a bearing on the development of the disease'.

Any cause of both the subfertility and the low offspring sex ratio (proportion male) would therefore seem a promising candidate also as a cause of the disease, so I should like to suggest one.

I have adduced very substantial quantities of data to support the hypothesis that the sexes of mammalian (and among them, human) offspring are partially dependent on the hormone levels of both parents around the time of conception (James, 1996). *Ex hypothesi*, low androgen levels in either parent are associated with the subsequent production of daughters. Moreover, levels of androgens (particularly dihydrotestosterone) in men are positively associated with coital rates (Dabbs and Morris, 1990; Mantzoros et al, 1995): and coital rates are powerfully and positively associated with fecundability, the probability of conceiving in a month at risk (Potter and Millman, 1986). Accordingly, I suggest that one cause of testicular cancer is suboptimal androgen levels. These, in turn, may be influenced by the intrauterine environment in which the patient gestated.

*WH James*
*The Galton Laboratory, University College London,*
*Wolfson House, 4 Stephenson Way, London NW1 2HE, UK*

**REFERENCES**

Dabbs JM and Morris R (1990) Testosterone, social class and antisocial behavior in a sample of 4462 men. *Psychol Sci* **1**: 1–3

Fosså SD and Kravdal Ø (2000) Fertility in Norwegian testicular cancer patients. *Br J Cancer* **82**: 737–741

James WH (1996) Evidence that mammalian sex ratios at birth are partially controlled by parental hormone levels at the time of conception. *J Theoret Biol* **180**: 271–286

Mantzoros CS, Georgiadis EI and Trichopoulos D (1995) Contribution of dihydrotestosterone to male sexual behaviour. *Br Med J* **310**: 1289–1291

Møller H (1998) Trends in sex ratio, testicular cancer and male reproductive hazards: are they connected? *Acta Pathol Microbiol Scand* **106**: 232–239

Potter RG and Millman SR (1986) Fecundability and the frequency of marital intercourse: new models incorporating the aging of gametes. *Population Stud* **40**: 159–170.

© 2000 Cancer Research Campaign

